# The treatment of transarterial chemoembolization/hepatic arterial infusion chemotherapy combined with lenvatinib and PD-1 inhibitor is effective against hepatocellular carcinoma with portal vein tumor thrombus: A systematic review

**DOI:** 10.3389/fonc.2023.1054072

**Published:** 2023-03-09

**Authors:** Gao Yuanren, Linan Yin, Ruibao Liu, Yali Cui

**Affiliations:** ^1^ Department of Interventional, Harbin Medical University Cancer Hospital, Harbin, China; ^2^ Department of Nuclear Medicine, Harbin Medical University Cancer Hospital, Harbin, China

**Keywords:** lenvatinib, PD-1 inhibitor, TACE, HAIC, liver cancer, portal vein tumor thrombus, efficacy, safety

## Abstract

**Background:**

Lenvatinib combined with programmed cell death protein-1 inhibitor has achieved good survival results in the treatment of hepatocellular carcinoma with portal vein tumor thrombus. Transarterial chemoembolization (TACE) or hepatic arterial infusion chemotherapy (HAIC) has attracted attention because of its high response rate and favorable survival rate in patients with liver cancer and portal vein tumor thrombus. This study aimed to compare the efficacy and safety of Lenvatinib combined with programmed cell death protein-1 inhibitor plus transarterial chemoembolization or hepatic arterial infusion chemotherapy in patients with hepatocellular carcinoma with portal vein tumor thrombus.

**Method:**

We searched PubMed, Embase and the Cochrane Library for studies. These included randomized controlled trials or clinical trials of Lenvatinib plus programmed cell death protein-1 inhibitor plus transarterial chemoembolization or hepatic arterial infusion chemotherapy (intervention group) versus Lenvatinib plus programmed cell death protein-1 inhibitor or Lenvatinib plus transarterial chemoembolization/hepatic arterial infusion chemotherapy or Lenvatinib alone (control group) in liver cancer with portal vein tumor thrombus The primary outcomes were overall survival and progression-free time, and the secondary outcomes were response rate and the rate of adverse events. According to the heterogeneity among different studies, Revman5.4 was used to conduct fixed effect or random effect model analysis.

**Results:**

Five clinical trials were included, including 311 cases in the intervention group and 309 cases in the control group. In terms of efficacy, compared with the control group, the overall survival (HR=1.88, 95%CI: 1.57-2.25, P < 0.00001) and progression-free survival (HR=1.62, 95%CI: 1.41-1.86, P < 0.00001), better efficacy, and better disease response than the control group. In terms of safety, the risk of treatment-related adverse events in the intervention group was higher than that in the control group, and White Blood cell count decreased (RR=0.72, 95%CI: 0.38-1.37, P=0.32), Platelet count decreased (RR=0.99, 95%CI: 0.65-1.51, P=0.96) and Total bilirubin increased (RR=0.86, 95%CI: Increased) 0.88-1.28, P=0.46) were lower than those in the control group, and the rest were higher than those in the control group, and the differences in some results were statistically significant.

**Conclusions:**

Transarterial chemoembolization or hepatic arterial infusion chemotherapy combined with Lenvatinib plus programmed cell death protein-1 inhibitor can effectively delay the progression, prolong the survival period and improve the quality of life of liver cancer patients with portal vein tumor thrombus.

## Introduction

Hepatocellular carcinoma (HCC) is a common malignant tumor with high mortality. Portal vein tumor thrombosis (PVTT) is a common clinical manifestation and an important poor prognostic factor of HCC, and up to 23% of HCC patients are complicated with PVTT (1). HCC patients with PVTT tend to develop rapidly, with a median overall survival (OS) of only about 2.7 months (1). Guidelines and expert consensus recommend surgery for some HCC patients with PVTT. However, the onset of HCC is insidious, and most of the patients have developed to the middle and late stage when they visit the hospital. Only about 20% of the patients are suitable for surgical treatment, while most of the other patients mainly receive palliative care such as systematic treatment, intervention and radiotherapy. At present, transcatheter arterial chemoembolization (TACE) and hepatic arterial infusion chemotherapy (HAIC) have been used to provide local disease control for patients with acceptable liver function and tumor burden, which can improve the survival rate of patients with advanced HCC and become the first choice of treatment for patients with HCC who cannot undergo surgery (2–4).

Lenvatinib, an oral, small-molecule, multi-target tyrosine kinase inhibitor, is the first-line drug for advanced HCC. At present, Lenvatinib has been proved effective and well tolerated in randomized phase III trials (5). Immune checkpoint inhibitors have shown promising clinical efficacy and safety in patients with advanced HCC. Considering the different anticancer mechanisms of TKIs, PD-1 inhibitors, and TACE or HAIC, combining these three modalities may show potential synergistic effects and promising initial efficacy outcomes in patients with hepatocellular carcinoma with portal vein tumor thrombus. Therefore, this study systematically evaluated the efficacy and safety of TACE or HAIC combined with Lenvatinib plus PD-1inhibitor in the treatment of hepatocellular carcinoma with portal vein tumor thrombus, in order to provide a basis for the rational use of this treatment model by clinicians.

## Materials and methods

### Search strategy and study selection

Eligible RCTs and clinical trials were searched from PubMed, Embase, and the Cochrane Central Registry of Controlled Trials through September 18, 2022. Search terms for these RCTs and clinical trials included: Lenvatinib, PD-1; Hepatic arterial infusion Chemotherapy; Transarterial Chemoembolization. HCC. Portal vein tumor Thrombus; RCT, clinical trials, etc. Additional studies were retrieved from the proceedings of the American Association for Cancer Research (AACR), Chinese Society of Clinical Oncology (CSCO), American Society of Clinical Oncology (ASCO), CHINA ANTI CANCER ASSOCIATION (CACA) and European Society for Medical Oncology (ESMO) to include more complete data.

We specify exclusion and inclusion criteria in advance. Eligible RCTs met the following inclusion criteria: patients with liver cancer with portal vein tumor thrombus confirmed by imaging or pathological diagnosis; The experimental group was treated with Lenvatinib+PD-1+TACE/HAIC, while the control group was treated with Lenvatinib+PD-1 or Lenvatinib+TACE/HAIC or Lenvatinib monotherapy. The outcome end points were OS, PFS, response rate, treatment-related adverse events, and laboratory-related adverse events. Studies were excluded based on the following criteria: designed as a retrospective or prospective observational cohort study; Lack of relevant data; In the form of comments, case reports, letters, reviews, editorials.

### Data extraction and quality assessment

Extraction items included: first author, year of publication, trial phase, number of patients, OS, PFS, response rate and treatment-related adverse events, and laboratory-related adverse events. We used the Cochrane bias Risk tool (6) to assess the methodological quality of the included trials. Two authors independently extracted data and conducted quality assessment in the process. If there was any discrepancy, a third party would resolve it.

### Statistical analysis

RevMan5.4 software was used for data analysis of the included studies. χ^2^ test and I^2^ statistic were used to assess heterogeneity. If I^2^ is greater than 50%, the random effects model is chosen, which implies significant heterogeneity, otherwise, the fixed effects model is used. The publication bias of the selected studies was assessed by funnel plots.

## Results

### Literature search

**Figure 1:**
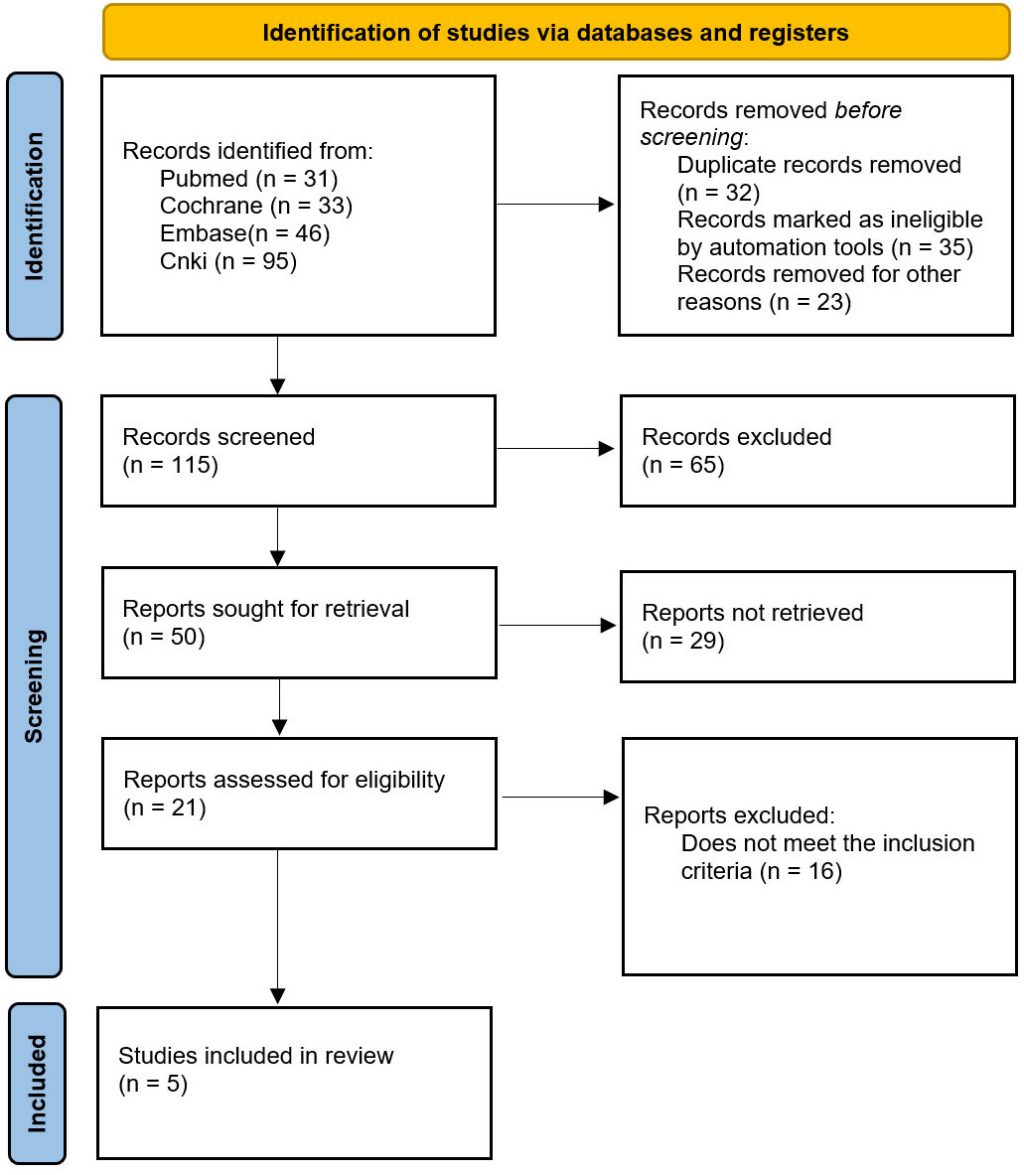
Literature screening process and results.

A total of 205 relevant studies were identified in the initial search strategy and screened according to the inclusion and exclusion criteria. The final quantitative analysis included 5 (7–11) randomized controlled trials and clinical trials involving 620 patients, as shown in **Figure 1**.

### Characteristics of the studies and quality assessment

**Figure 2:**
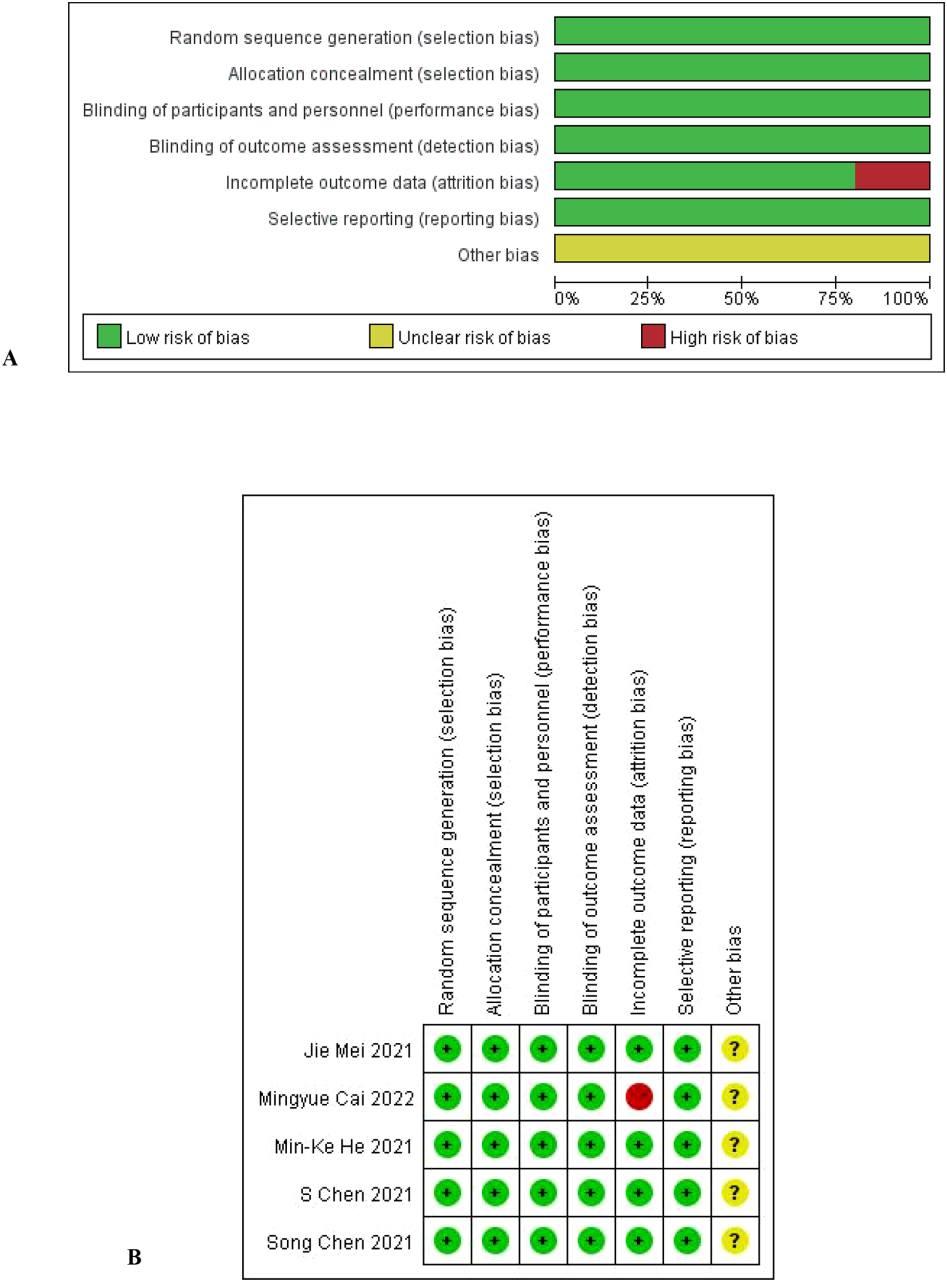
**(A, B)** Evaluation results of methodology quality of included studies.

The literature characteristics are shown in **Table 1**, and the quality assessment results are shown in **Figures 2A, B**.

**Table 1:** Basic characteristics of included studies and main evaluation indicators (7–11).

First Author	Year	NO. of Patients with experimental	NO. of Patients with Control	protocol of Patients with experimental	protocol of Patients with control	HR for OS [95% CI]	p-Value for OS	HR for PFS [95% CI]	p-Value for PFS	CR (E/C)	PR (E/C)	SD (E/C)	PD (E/C)	ORR (E/C)	DCR (E/C)
Jie Mei (7)	2021	45	25	LEN+PD-1+HAIC	LEN+PD-1	0.60[0.43-0.83]	0.0015	0.74[0.55-0.98]	0.032	0/0	18/4	20/7	5/6	18/4	38/11
Mingyue Cai (8)	2022	41	40	LEN+PD-1+TACE	LEN+TACE	NA	NA	NA	NA	3/1	14/5	9/3	4/2	23/21	35/32
Song Chen (9)	2021	70	72	LEN+PEM+TACE	LEN+TACE	0.56[0.38-0.83]	0.004	0.60[0.39-0.91]	0.006	7/4	26/16	16/18	18/30	33/20	49/38
S Chen (10)	2021	84	86	LEN+PEM+HAIC	LEN+PEM	0.52[0.36-0.75]	0.001	0.61[0.43-0.85]	0.001	13/8	37/28	24/35	9/12	50/36	0/0
Min-Ke He (11)	2021	71	86	LEN+TOR+HAIC	LEN	0.40[0.24-0.66]	0.001	0.48[0.33-0.70]	0.001	10/0	38/14	16/48	7/24	48/14	64/62

CR, complete remission; PR, partial remission; SD, stable disease; PD, progressive disease; ORR, Overall response rate; DCR, Disease control rate; TACE, transcatheter arterial chemoembolization; HAIC, Hepatic artery infusion chemotherapy; PD-1, programmeddeath-1; OS, overall survival; PFS, progression-free survival; LEN, Lenvatinib; PEM, pembrolizumab; HR, Hazard ratio; NA, Not mentioned; E/C, experimental/control.

### Primary analysis

#### Efficacy

A total of 5 studies reported data on validity and response rate, with no statistical heterogeneity (I^2^ < 50%). Fixed-effect model was used for analysis, whereas random effect model was used for analysis. The results are as follows:

#### Overall survival and progression-free survival

**Figure 3:**
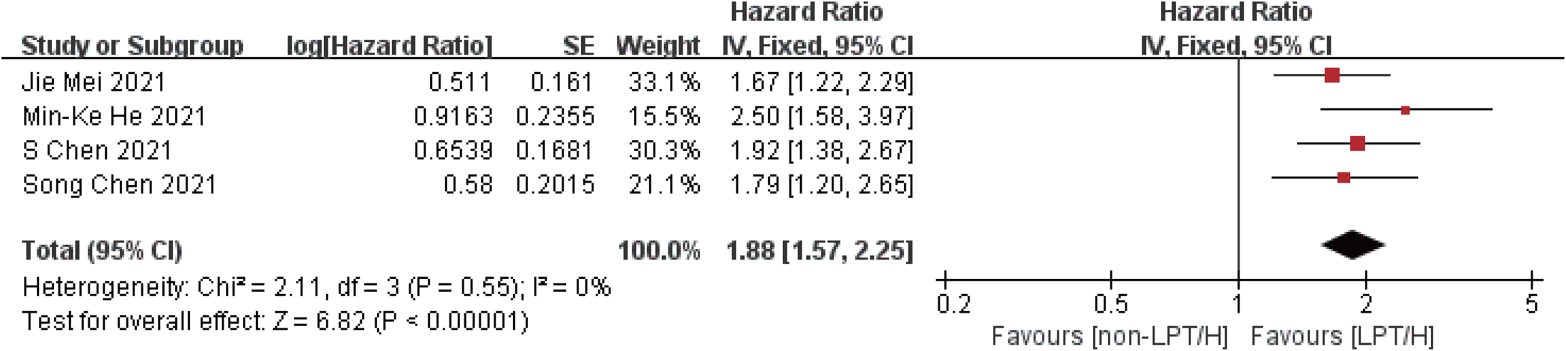
Meta-analysis results of OS.

**Figure 4:**
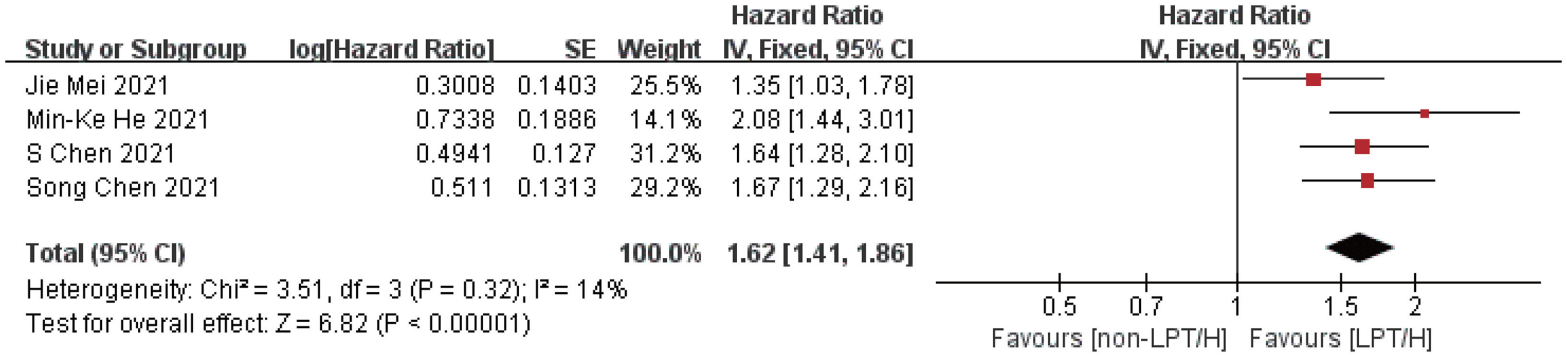
Meta-analysis results of PFS.

Compared with the control group, the intervention group showed longer overall survival (HR = 1.88, 95%CI: 1.57-2.25, P < 0.00001) and progression-free period (HR = 1.62, 95%CI: 1.41-1.86, P < 0.00001), indicating that the overall survival of the intervention group was 1.88 times longer than that of the control group, and the progression-free period was 1.62 times longer than that of the control group. The efficacy of the intervention group was better than that of the control group, as shown in **Figures 3**, **4**.

#### Response outcomes

Compared with the control group, the intervention group achieved higher complete remission rate (CR) (RR = 2.61, 95%CI: 1.43-4.75, P=0.002) and partial remission rate (PR) (RR = 2.05, 95%CI: 1.43-4.75, P=0.002), respectively. 1.39-3.04, P=0.0003), stable disease rate (SD) (RR = 0.90, 95%CI: 0.52-1.56, P=0.71), progressive disease (PD) rate (RR = 0.58, 95%CI: 0.42-0.82, P=0.002) and Overall response rate (ORR) (RR = 1.85, 95%CI: 1.16-2.94, P=0.01) and Disease control rate (DCR) (RR = 1.27, 95%CI: 1.06-1.52, P=0.009). The above results suggest that the intervention group has a higher disease remission rate and disease control rate, as well as a lower disease progression rate in the treatment of liver cancer with portal vein tumor thrombus, as shown in **Table 2**.

**Table 2:** Response outcomes.

Outcomes	Studies	Participants	Heterogeneity test results		Effect model	Meta-analysis results	
			*P*	*I^2^*		95%CI	*P*
CR	4	550	0.26	25%	Fixed	2.61 [1.43, 4.75]	0.002
PR	5	620	0.08	52%	Random	2.05 [1.39, 3.04]	0.0003
SD	5	620	0.003	75%	Random	0.90 [0.52, 1.56]	0.71
PD	5	620	0.36	8%	Fixed	0.58 [0.42, 0.82]	0.002
ORR	5	620	0.0005	80%	Random	1.85 [1.16, 2.94]	0.01
DCR	4	450	0.08	55%	Random	1.27 [1.06, 1.52]	0.009

CR, complete remission; PR, partial remission; SD, stable disease; PD, progressive disease; ORR, Overall response rate; DCR, Disease control rate.

Among the above results, individual results (PR, SD, ORR, DCR) have statistical heterogeneity (I^2^ > 50%). We found that the greater heterogeneity may be caused by different interventions in the control group, so according to the different interventions in the control group, subgroup analysis was carried out to eliminate heterogeneity and ensure the reliability of the results. The results show that different interventions in the control group affect the stability of the results. After subgroup analysis, the heterogeneity of the results obtained by dividing the control group into LEN + PD-1 and LEN + TACE subgroups is small (I^2^ < 50%), which shows that the heterogeneity comes from different interventions in the control group, as shown in **Table 3**.

**Table 3:** Response outcomes - Subgroup analysis.

Outcomes	Interventions in the control group	Studies	Participants	Heterogeneity test results		Effect model	Meta-analysis results	
				*P*	*I^2^*		95%CI	*P*
PR	LEN+PD-1	2	240	0.24	28%	Fixed	1.53 [1.07, 2.20]	0.02
	LEN+TACE	2	223	0.36	0%	1.93 [1.22, 3.05]	0.005
SD	LEN+PD-1	2	240	0.05	33%	Fixed	0.88 [0.62, 1.27]	0.50
	LEN+TACE	2	223	0.09	35%	1.21 [0.72, 2.03]	0.47
ORR	LEN+PD-1	2	240	0.62	0%	Fixed	0.20 [0.08, 0.32]	0.001
	LEN+TACE	2	223	0.25	25%	0.13 [-0.02, 0.28]	0.08
DCR	LEN+PD-1	2	227	0.36	0%	Fixed	4.61 [2.27, 9.37]	0.0001
	LEN+TACE	2	223	0.60	0%	1.90 [1.05, 3.44]	0.03

PR, partial remission; SD, stable disease; ORR, Overall response rate; DCR, Disease control rate; TACE, transcatheter arterial chemoembolization; PD-1, programmeddeath-1; LEN, Lenvatinib.

### Safety

#### Treatment-related AEs

A total of five studies reported data on treatment-related adverse events. Among all treatment-related adverse events, compared with the control group, Pruritus (RR = 1.71, 95%CI: 0.83-3.53, P=0.14) and Pain (RR = 1.27, 95%CI: 0.87-1.84, P=0.22), Fever (RR = 1.36, 95%CI: 0.82-2.24, P=0.23), Diarrhea (RR = 1.13, 95%CI: 0.82-1.56, P=0.46), Decreased appetite (RR = 1.42, 95%CI: 0.88-2.30, P=0.15), Hypothyroidism (RR = 1.32, 95%CI: 0.82-2.12, P=0.26) and Hyperthyroidism (RR = 1.67, 95%CI: 0.22-12.52, P=0.62) had higher risk ratios, but the difference was not significant. Nausea was Nausea only in Rash (RR = 1.73, 95%CI: 1.08-2.77, P=0.02), Fatigue (RR = 1.53, 95%CI: 1.17-2.01, P=0.002) and Nausea (RR = 1.82, 95%CI: 1.26-2.62, P=0.001), as shown in **Table 4**. The above results showed that the incidence of treatment-related adverse events of liver cancer with portal vein tumor thrombus in the intervention group was higher than that in the control group, and the difference in some adverse events was significant. In clinical practice, attention should be paid to the adverse reactions caused by the drug to ensure the safety of treatment.

**Table 4:** Meta-analysis results of Treatment-related AEs.

Outcomes	Studies	Participants	Heterogeneity test results		Effect model	Meta-analysis results	
			*P*	*I^2^*		95%CI	*P*
Rash	5	620	0.29	19%	Fixed	1.73 [1.08, 2.77]	0.02
Pruritus	4	463	0.83	0%	Fixed	1.71 [0.83, 3.53]	0.14
Pain	4	478	0.17	41%	Fixed	1.27 [0.87, 1.84]	0.22
Fever	2	240	0.97	0%	Fixed	1.36 [0.82, 2.24]	0.23
Diarrhea	5	620	0.83	0%	Fixed	1.13 [0.82, 1.56]	0.46
Fatigue	5	620	0.51	0%	Fixed	1.53 [1.17, 2.01]	0.002
Nausea	5	620	0.45	0%	Fixed	1.82 [1.26, 2.62]	0.001
Decreased appetite	4	463	0.82	0%	Fixed	1.42 [0.88, 2.30]	0.15
Hypothyroidism	5	620	0.81	0%	Fixed	1.32 [0.82, 2.12]	0.26
Hyperthyroidism	2	251	0.62	0%	Fixed	1.67 [0.22, 12.52]	0.62

#### Laboratory-related AEs

A total of five studies reported data on laboratory-related adverse events. Among all laboratory-related adverse events, levels of Neutropenia (RR = 2.44, 95%CI: 1.55-3.84, P=0.0001) and Alanine aminotransferase levels increased in the intervention group compared with the control group (RR = 1.53, 95%CI: 0.83-2.81, P=0.17), Aspertate aminotransferase increased (RR = 1.66, 95%CI: 0.91-3.02, P=0.10), and Albumin decreased (RR = 3.30, 95%CI: 0.28-39.00, P=0.34), and the difference was significant only in Neutropenia. In addition, decreased in White Blood cell count (RR = 0.72, 95%CI: 0.38-1.37, P=0.32), decreased in the White Blood cell count (RR = 0.99, 95%CI: 0.65-1.51, P=0.96) and the levels of Total bilirubin increased (RR = 0.86, 95%CI: 0.58-1.28, P=0.46) were lower than those in the control group, with no significant differences, as shown in **Table 5**. The above results showed that the incidence of some laboratory-related adverse events of liver cancer with portal vein tumor thrombus in the intervention group was higher than that in the control group, and the difference in some adverse events was significant.

**Table 5:** Meta-analysis results of Laboratory-related AEs.

Outcomes	Studies	Participants	Heterogeneity test results		Effect model	Meta-analysis results	
			*P*	*I^2^*		95%CI	*P*
White blood cell count decreased	2	240	0.58	0%	Fixed	0.72 [0.38, 1.37]	0.32
Platelet count decreased	3	321	0.58	0%	Fixed	0.99 [0.65, 1.51]	0.96
Neutropenia	3	308	0.29	20%	Fixed	2.44 [1.55, 3.84]	0.0001
Alanine aminotransferase increased	5	620	0.0001	84%	Random	1.53 [0.83, 2.81]	0.17
Aspertate aminotransferase increased	5	620	0.0001	84%	Random	1.66 [0.91, 3.02]	0.10
Total bilirubin increased	3	321	0.70	0%	Fixed	0.86 [0.58, 1.28]	0.46
Albumin decreased	3	397	0.0001	93%	Random	3.30 [0.28, 39.00]	0.34

Among the above results, individual results (Alanine aminotransferase, Aspertate aminotransferase, Albumin decreased) have statistical heterogeneity (I^2^ > 50%). We found that the greater heterogeneity may be caused by different interventions in the control group, so according to the different interventions in the control group, subgroup analysis was carried out to eliminate heterogeneity and ensure the reliability of the results. The results show that different interventions in the control group affect the stability of the results. After subgroup analysis, the heterogeneity of the results obtained by dividing the control group into LEN+PD-1 and LEN + TACE or LEN subgroups is small (I^2^ < 50%), which shows that the heterogeneity comes from different interventions in the control group, as shown in **Table 6**.

**Table 6:** Meta-analysis results of Laboratory-related AEs - Subgroup analysis.

Outcomes	Interventions in the control group	Studies	Participants	Heterogeneity test results		Effect model	Meta-analysis results	
				*P*	*I^2^*		95%CI	*P*
Alanine aminotransferase increased	LEN+PD-1	2	240	0.02	81%	Random	1.47 [0.48, 4.51]	0.50
	LEN+TACE	2	223	0.41	0%	1.15 [0.68, 1.94]	0.61
Aspertate aminotransferase increased	LEN+PD-1	2	240	0.05	74%	Fixed	1.12 [0.65, 1.92]	0.68
	LEN+TACE	2	223	0.78	0%	1.65 [0.86, 3.17]	0.13
Albumin decreased	LEN+PD-1	2	240	0.39	0%	Fixed	0.94 [0.47,1.88]	0.86
	LEN	2	157	—	—	—	—

TACE, transcatheter arterial chemoembolization; PD-1, programmeddeath-1; LEN, Lenvatinib. -, not available.

## Sensitivity analysis and publication bias assessment

**Figure 5:**
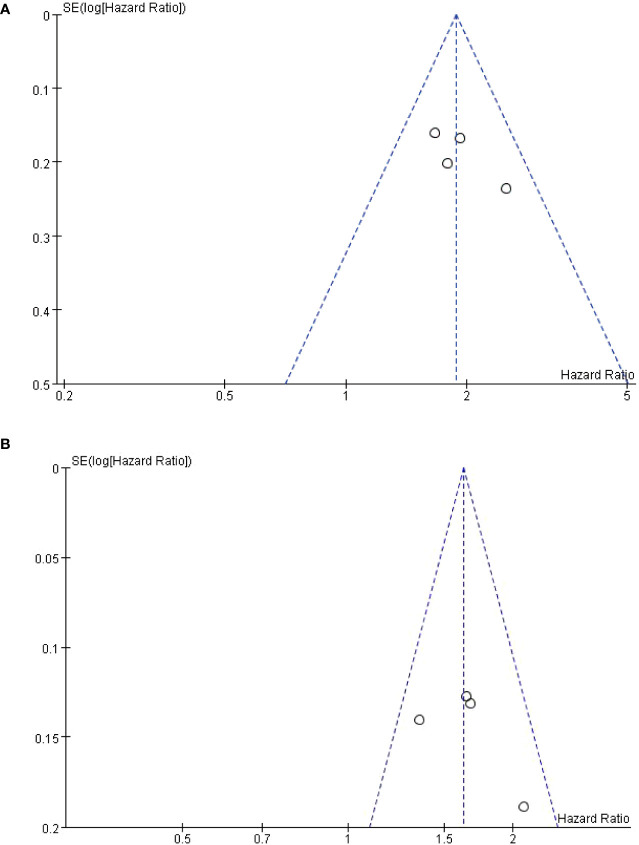
Inverted funnel plot of OS **(A)** and PFS **(B)**.

Publication bias was assessed only in OS and PFS. Funnel plots are symmetric, indicating no significant publication bias, see **Figures 5A, B**. The sensitivity analysis of the results, and the meta-analysis ignoring each study in turn, showed that no significant change was found, indicating that the results of this study were stable.

## Discussion

Liver cancer is one of the most common malignant tumors in the world, and the incidence of HCC complicated with Portalvein tumor thrombus (PVTT) is 44%-62.2%, which is an important factor affecting the prognosis of advanced liver cancer (12). The median survival time of HCC patients with PVTT is only 2.7 months, which has become an important factor seriously affecting the survival time of HCC patients. Currently, Transcatheter arterial chemoembolization (TACE) and hepatic arterial infusion Chemotherapy (hepatic arterial infusion), HAIC is the main method for the treatment of advanced liver cancer, but the effect on portal vein tumor thrombus is not as good as expected. In recent years, interventional therapy based on TACE or HAIC has become more mature and its efficacy has been widely recognized (13). Therefore, clinical attempts have been made to explore the combination therapy based on TAC or HAICE to improve the survival time of patients with hepatocellular carcinoma with portal vein tumor thrombus. A meta-analysis of the retrospective studies of TACE plus sorafenib has shown that the combined treatment group has higher Objective remission rate (ORR) and Disease control rate (DCR), Moreover, the Time to tumor progression (TTP) was also significantly prolonged. Subgroup analysis showed that the effect of combined treatment on the primary and lower branches of portal vein was more obvious than that of liver cancer with portal vein main tumor thrombus (14). However, there are also a series of problems in the clinical application of sorafenib, such as serious adverse reactions, heavy economic burden and the emergence of drug resistance in long-term use of liver cancer cells. In Zheng et al. ‘s study on TACE combined with sorafenib in the treatment of advanced liver cancer, 6.7% of patients developed liver insufficiency and 24.1% died of liver failure (15).

Lenvatinib belongs to a quinoline carboxyamide class of antitumor drugs, tyrosine kinase inhibitors. It in for inhibition of tumor diseases, mainly through anti-angiogenesis reaction to implement, can achieve the fibroblast growth factor receptor (FGFR) 1-4, endothelial growth factor receptor (VEGFR) - 3 receptors, such as the effective control, which can help get block new blood vessels form, so as to realize the tumor microenvironment of vascular permeability effectively reduce. Clinically, hepatic arterial chemoembolization (TACE) combined with Lenvatinib targeted therapy for advanced hepatocellular carcinoma can control the disease progression, reduce the levels of tumor vascular factors and tumor markers, and stabilize the liver function of patients. Immune checkpoint inhibitors, including programmed death 1 (PD-1) and programmed death ligand 1 (PD-L1) inhibitors, have demonstrated relatively excellent clinical effects in patients with advanced HCC. Although the phase III trial of immune checkpoint inhibitor monotherapy failed to achieve its primary survival endpoint (16), other studies have shown that immune checkpoint inhibitors combined with antiangiogenic agents have a prominent clinical effect in the treatment of advanced HCC (17). Currently, Lenvatinib is used in combination with transarterial chemoembolization (TACE) or hepatic arterial infusion Chemotherapy. HAIC and programmed cell death protein-1 (PD-1) mab have been gradually used in the treatment of hepatocellular carcinoma with portal vein tumor thrombus, but there is no systematic evaluation of the efficacy and safety of the combination of HAIC and PD-1 inhibitor mab. This study comprehensively evaluated the efficacy and safety of combined treatment of hepatocellular carcinoma with portal vein tumor thrombus based on published clinical trials or randomized controlled trials, so as to provide a scientific basis for clinical practice.

This study conducted meta-analysis on various independent research results to discuss the therapy of Lenvatinib combined with PD-1 inhibitor plus transarterial Chemoembolization/hepatic arterial infusion Chemotherapy in patients with hepatocellular carcinoma (HCC) with portal venous tumor emboli. A total of 5 studies were included in this study. The primary outcome measures included OS, PFS and clinical response rate, and the risk of treatment-related adverse events and laboratory-related adverse events caused by them were analyzed. The results of this meta-analysis showed that in terms of effectiveness, compared with the control group, the intervention group had overall survival (HR = 1.88, 95%CI: 1.57-2.25, P < 0.00001) and progression-free period (HR = 1.62, 95%CI: 1.41-1.86, P < 0.00001), and the results were statistically different. In addition, the response rates of the intervention group were higher, with the complete response rate reaching 2.61 times of the control group and the partial response rate reaching 2.05 times of the control group. The disease stabilization rate and disease progression rate were 0.90 and 0.58 times of the control group, respectively. According to the current meta-analysis results, the effectiveness of the intervention group in the treatment of liver cancer with portal vein tumor thrombus is better than that of the control group. In terms of safety, the intervention group showed a lower safety profile. The main treatment-related adverse events include Rash, Pruritus, Pain, Fever, Diarrhea, Fatigue, Nausea, Decreased appetite, Hypothyroidism and Hyperthyroidism. Among these adverse events, Nausea (RR = 1.82, 95%CI: 1.26-2.62, P=0.001), Rash (RR = 1.73, 95%CI: 1.08-2.77, P=0.02), and Pruritus (RR = 1.71, 95%CI: 0.83-3.53, P=0.14), and other adverse events were higher than those in the control group. Inthe laboratory related adverse events mainly include White Blood cell count decreased, Platelet count decreased, Neutropenia, Alanine aminotransferase Increased, aspertate aminotransferase increased, Total bilirubin increased, and Albumin decreased. Among the above adverse events, Neutropenia (RR = 2.44, 95%CI: 1.55-3.84, P=0.0001) and Alanine aminotransferase levels increased (RR = 1.53, 95%CI: 0.83-2.81, P=0.17), aspertate aminotransferase increased (RR = 1.66, 95%CI: 0.91-3.02, P=0.10), and Albumin decreased (RR = 3.30, 95%CI: 0.28-39.00, P=0.34) were higher than those in the control group, and the rest were lower than those in the control group. These adverse events should be noted clinically for symptomatic treatment. Thus, we evaluated the publication bias of this study by funnel plot, which preliminarily suggested that there was no significant publication bias, and the results of sensitivity analysis suggested that the results of this meta-analysis were stable.

Due to the late launch of Lenvatinib, limited original studies that could be included and data that could be extracted, this study could not conduct further subgroup analysis, and there may also be loss to follow-up bias in the original study. All these factors may affect the accuracy of meta-analysis results, so this study has some limitations. The evaluation results need to be further verified by independent clinical trials. In the future, researchers will carry out further large-scale, high quality research trials which could provide clear transarterial chemoembolization/hepatic arterial infusion the Chemotherapy combined with lenvatinib plus PD-1 inhibitors treatment strategy will provide a more reliable basis for the clinical prognosis, risk stratification and medication guidance in patients with hepatocellular carcinoma with portal vein tumor thrombus.

## Data availability statement

The original contributions presented in the study are included in the article/Supplementary Material. Further inquiries can be directed to the corresponding authors.

## Author contributions

GY and LY: Searched the database and analysed the data. LY and YC: Elected the study and extracted the data. GY: Wrote the manuscript. RL: Reviewed the manuscript. All authors contributed to the article and approved the submitted version.
